# Plasma growth differentiation factor-15 is an independent marker for aggressive disease in endometrial cancer

**DOI:** 10.1371/journal.pone.0210585

**Published:** 2019-01-15

**Authors:** Hilde Engerud, Kirsten Hope, Hege Fredriksen Berg, Kristine Eldevik Fasmer, Ingvild Løberg Tangen, Ingfrid Salvesen Haldorsen, Jone Trovik, Camilla Krakstad

**Affiliations:** 1 Centre for Cancer Biomarkers CCBIO, Department of Clinical Science, University of Bergen, Bergen, Norway; 2 Department of Obstetrics and Gynecology, Haukeland University Hospital, Bergen, Norway; 3 Department of Radiology, Haukeland University Hospital, Bergen, Norway; 4 Section for Radiology, Department of Clinical Medicine, University of Bergen, Bergen, Norway; National Cancer Center, JAPAN

## Abstract

**Objective:**

Better biomarkers are needed in order to identify patients with endometrial carcinoma at risk of recurrence and who may profit from a more aggressive treatment regimen. Our objective was to explore the applicability of plasma growth differentiation factor 15 (GDF-15) as a marker for recurrent disease, as well as a marker for poor prognosis and lymph node metastases.

**Methods:**

EDTA-blood samples were obtained from 235 patients with endometrial cancer before primary surgery. For 36 of these patients, matching blood samples were collected at time of recurrence. Blood samples were also collected from 78 patients with endometrial hyperplasia. Plasma GDF-15 was measured by an enzyme-linked immunosorbent assay (ELISA). Preoperative pelvic MRI scans for 141 patients were investigated in parallel for imaging variables.

**Results:**

Preoperative plasma level of GDF-15 was significantly higher for patients who experienced recurrence (1780 ng/L; 95% CI; 518–9475 ng/L) than for patients who did not develop recurrent disease (1236 ng/L; 95% CI; 307–7030 ng/L) (p<0.001). Plasma levels of GDF-15 at recurrence (2818 ng/L, 95% CI 2088–3548 ng/L) were significantly higher than plasma levels of GDF-15 measured at time of primary diagnosis (1857 ng/L, 95% CI; 1317–2398 ng/L) (p = 0.001). High plasma level GDF-15 independently predicts recurrent disease (OR = 3.14; 95% CI 2.10–4.76) and lymph node metastases (OR = 2.64; 95% CI 1.52–4.61). Patients with high plasma level of GDF-15 had significantly larger tumor volume (p = 0.008).

**Conclusion:**

Elevated plasma level of GDF-15 is associated with aggressive disease and lymph node metastasis in endometrial carcinoma. GDF-15 may be helpful in indicating recurrent disease.

## Introduction

Endometrial cancer is the most common gynecologic malignancy and the fourth most common cancer among women in industrialised countries. Incidence is increasing worldwide, mostly due to the obesity epidemic. The prognosis is good with an overall 5-year survival of 80%, however 15–20% of patients with a presumed low risk disease experience recurrence [1]. Better biomarkers are thus needed in order to identify patients at high risk of recurrence who may profit from a more aggressive treatment regimen. To date there are no biomarkers for prognosis routinely available or in widespread use in the clinic and the majority of suggested markers have been developed for immunohistochemistry based detection in patient tissue biopsies. However, there has been little focus on identifying markers in preoperative blood samples [2–4]. Such markers are less invasive than those from biopsy, easily obtainable and could also be measured repeatedly during the course of the disease. A robust prognostic plasma biomarker would therefore potentially be highly valuable in the clinic.

Growth differentiation factor-15 (GDF-15), is a distant member of the transforming growth factor (TGF)-beta superfamily, also named macrophage-inhibitory cytokine -1 (MIC-1), and was originally identified in activated macrophages [5]. The TGF-beta superfamily has a role in regulating inflammatory and apoptotic pathways in injured tissues and during disease processes. GDF-15 is associated with cell cycle arrest and apoptosis [6]. Expression is dramatically increased in diseased states, such as acute injury, inflammation and cancer [7]. The prognostic value of GDF-15 is previously explored in cardiac disease, during pregnancy and in cancer. It has been suggested as a prognostic biomarker where increased GDF-15 is associated with increased risk of death at 1 year in patients with non-ST-elevation acute coronary syndrome [8] as well as in patients with ST-segment elevation and myocardial infarction [9]. In the placenta GDF-15 is physiologically highly expressed [10] and low expression of GDF-15 is associated with miscarriages [11]. Elevated levels, however, are associated with diabetes mellitus and preeclampsia [12]. In cancer, GDF-15 overexpression has been reported in malignant melanomas, prostate-, pancreatic- and colonic cancers [13–16]. Furthermore, elevated serum levels of GDF-15 are linked to cancer-associated anorexia and weight loss in prostate cancer [17]. In gynecological malignancies GDF-15 is reportedly an independent marker of aggressive disease in ovarian cancer [18]. For uterine sarcomas, elevated GDF-15 may aid in discriminating aggressive sarcomas from benign leiomyomas [19], whereas for endometrial cancer increased GDF-15 expression has been reported to predict lymph node metastases and poor survival [20].

In the present study we wanted to explore the applicability of GDF-15 in predicting endometrial carcinoma recurrence. In addition, using an extensive panel of clinicopathological variables including survival, our aim was to validate GDF-15 as a prognostic marker in endometrial carcinoma and as a possible predictor of lymph node metastases.

## Materials and methods

### Patient samples

EDTA-blood samples were obtained preoperatively from 235 patients with endometrial cancer before primary surgery. During time of follow-up 48 patients developed recurrence and blood samples were collected from 36 of these patients at time of recurrence. In addition, EDTA-blood was collected from 78 patients diagnosed with endometrial hyperplasia. All patients have been diagnosed at Haukeland University Hospital, Norway between 2003 and 2014 and clinical data as well as blood samples were prospectively collected. Patients signed informed consent. Regional Committees for Medical and Health Research Ethics approval: 2009/2315 and 2014/1907. The median follow-up in this cohort is 43 months (range 1–189). Blood samples were centrifuged at 1600 g for 15 min and the plasma was stored at– 80 °C until measurement of GDF-15. Distribution of measured GDF-15 plasma level was not influenced by storage time.

Additionally, data regarding GDF-15 plasma levels were also locally available from an independent cohort of 466 endometrial cancer patients previously published [20]. In order to improve statistical power when predicting lymph node metastases and recurrence, the data were merged together with the current cohort when performing regression analyses. Data were missing for recurrence from 47 patients, histological type from 47 patients and myometrial infiltration from 49 patients, and the resulting cohort included 603 patients. Regarding lymph node status, data were missing for 190 patients and preoperative histology missing for 65 patients, and the cohort included 495 patients.

### GDF-15 measurements

GDF-15 in plasma was measured by the Human enzyme linked GDF-15 Quantakine ELISA kit (#DGD150, batch #P153423, R&D Systems, Minneapolis, USA). The ELISA was performed according to the manufacturer´s instructions. Briefly, 50 μL plasma sample or standard was added in a 96-well microplate coated with a monoclonal antibody specific for human GDF-15, and incubated for 2h in room temperature. Following washing, 200 μL human GDF-15 conjugate was added and incubated for 1 hour in room temperature. The wells were washed again before 200 μL of substrate solution were added and incubated for 30 min in room temperature protected from light, followed by 50 μL of stop solution. The absorbance was measured in a microplate reader at the wavelength of 450 nm, and plasma concentration of GDF-15 calculated. To confirm reproducibility, a subset (n = 102) were measured in duplicates. Clinical data were blinded while performing and evaluating laboratory investigations. The assay has a detection limit of 20 ng/L, an intraassay imprecision of 10.6% or less, and an interassay imprecision of 12.2% or less [21].

### Preoperative magnetic resonance imaging (MRI)

In parallel, preoperative pelvic MRI scans for 141 patients were assessed to derive the following imaging variables: endometrial tumor size, signs of deep myometrial invasion, cervical stroma invasion and lymph node metastases. MRI was conducted on a whole body 1.5-T MRI system (Siemens Avanto running Syngo v. B17, Erlangen, Germany) using a six channel body coil applying a standardized imaging protocol [22]. To reduce motion artefacts 20 mg of butylscopolamine bromide (Buscopan; Boehringer, Ingelheim, Germany) was administered intravenously. Mean time (range) between MRI examination and surgical staging was 1.5 (0–12) weeks.

### Statistical analyses

Statistical analyses were conducted applying Statistical Program for the Social Sciences (SPSS), version 24 (IBM Inc. Chicago, IL, USA). All p-values were two sided and p-value of less than 0.05 was considered statistically significant. Pearson Chi-square or Fisher exact test were used for categorical data. Univariate survival analysis was performed using the Kaplan-Meier method and log-rank test, grouping low versus high concentration. Cut-off values for categorization were based on tertiles according to the size of the subgroups and the number of events in each category. The two lower GDF-15 tertiles were merged due to similar survival. Cut-off value based on this method was found to be near identical to our previous study [20] (cut-off in previously published cohort: 1400 ng/L, cut-off in this cohort 1418 ng/L). For analyses where the cohorts were merged, cut-off value was 1418 ng/L. Low-risk patients were defined as endometrioid histologic type and grade 1 and 2 disease, and high risk patients as endometrioid grade 3 and non-endometrioid. Disease-specific survival was defined as time from primary treatment to death from endometrial cancer. Patients who died from other causes or were lost to follow-up were censored at the date of death/last follow-up. Non-parametric tests Mann Whitney U or Wilcoxon Signed Rank were used for comparison of continuous data between study groups. Binary logistic regression was used to evaluate the odds ratio (OR) for lymph node metastases and recurrence.

## Results

### Plasma GDF-15 associates with poor survival, also in low-risk patients

**Fig 1:**
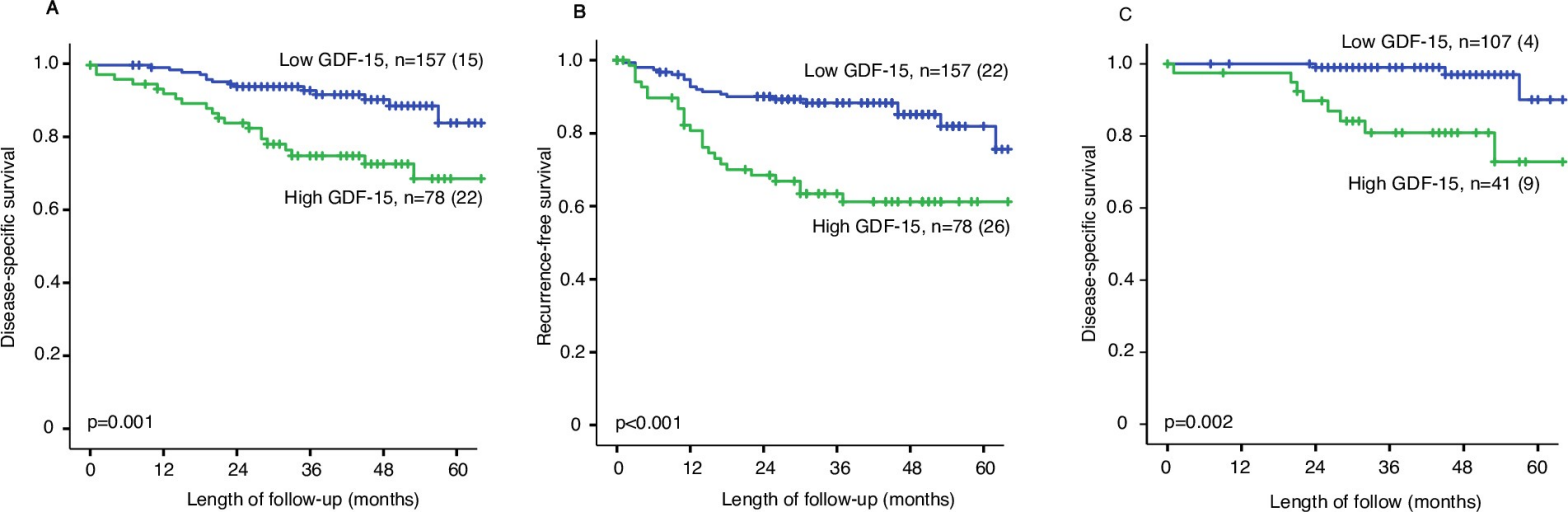
Disease-specific survival (A) and recurrence-free survival (B) in 235 patients illustrated by Kaplan Meier curves. Disease-specific survival in patients with a putative low-risk disease preoperatively, that is endometrioid grade 1 or 2 (C). P-values are calculated by the Mantel-Cox log rank test. Low GDF-15 is the two lower tertiles, 1. and 2. combined. High GDF-15 is the 3. tertile. Number of cases are given in each category and in parenthesis number of disease-specific deaths or recurrence.

To validate previous observations that plasma level of GDF-15 is a biomarker for poor prognosis in endometrial cancer, plasma level of GDF-15 was determined in an independent patient population including 235 patients with primary endometrial carcinoma. High level of plasma GDF-15 was associated with reduced disease-specific survival (p = 0.001, Fig 1A) with 5-year survival rate of 72.9% compared to 94.1% in patients with low GDF-15. In addition, high GDF-15 indicated reduced recurrence-free survival (p<0.001, Fig 1B) with 5-year recurrence-free survival rate of 61.2% compared to 89.3% in endometrial cancer. GDF-15 level was significantly higher in patients aged >66 years, and in patients with advanced FIGO stage, non-endometrioid histologic subtype, high grade and with deep myometrial infiltration (all p-values ≤0.003, Table 1). These findings are in line with previous findings from a separate cohort from our hospital [20]. To further explore the usefulness of plasma GDF-15, we performed analyses in the low-risk subgroup of patients, defined by endometrioid histology and grade 1 or 2 disease on preoperative curettage specimen (n = 148). Also in this patient subgroup, high level of plasma GDF-15 was associated with poor prognosis (p = 0.002, Fig 1C) with a 5-year survival rate of 72.8% compared to 97% in patients with low GDF-15. High age, high grade and deep myometrial infiltration were all associated with high levels of GDF-15 (all p-values ≤0.007, Table 2) in low-risk patients. BMI did not correlate with increasing plasma concentrations of GDF-15 (R^2^ 0.003).

**Table 1:** GDF-15 measured in plasma samples from 235 patients with endometrial cancer in relation to clinicopathological factors.

	Low, n (%)	High, n (%)	P-value*
**Age, y**			<0.001
<66	86 (84)	16 (16)	
≥66	71 (53)	62 (47)	
**FIGO**			0.003
I/II	140 (71)	58 (29)	
III/IV	17 (46)	20 (54)	
**Histologic type**			0.002
Endometrioid	126 (72)	48 (28)	
Non-endometrioid	31 (51)	30 (49)	
**Non-endometrioid types**			0.003
Clear cell	4 (40)	6 (60)	
Serous	20 (61)	13 (39)	
Carcinosarcomas	3 (25)	9 (75)	
Undifferentiated	4 (67)	2 (33)	
Histologic grade**			0.002
Grade 1	76 (84)	15 (16)	
Grade 2	27 (55)	22 (45)	
Grade 3	18 (64)	10 (36)	
**Myometrial infiltration**			0.001
<50%	101 (75)	33 (25)	
≥50%	55 (56)	44 (44)	

*P-values are calculated by Chi-Square test or Fisher exact test.

**Endometrioid included only.

Low = 1. and 2. tertile

High = 3. tertile

FIGO: International Federation of Gynecology and Obstetrics

**Table 2:** GDF-15 in plasma samples in relation to clinicopathological factor in patients with preoperative low-risk staging*.

	Low, n (%)	High, n (%)	P-value^#^
**Age, years**			0.005
<66	59 (83)	12 (17)	
≥66	48 (62)	19 (38)	
**FIGO**			0.36
I/II	98 (74)	35 (26)	
III/IV	9 (60)	6 (40)	
**Histologic type**			0.218
Endometrioid	103 (74)	37 (26)	
Non-endometrioid	4 (50)	4 (50)	
**Non-endometrioid types**			0.242
Clear cell	0	1 (100)	
Serous	3 (50)	3 (50)	
Carcinosarcomas	1 (100)	0	
Histologic grade**			0.007
Grade 1	70 (82)	15 (18)	
Grade 2	24 (57)	18 (43)	
Grade 3	5 (56)	4 (44)	
**Myometrial infiltration**			0.007
<50%	73 (80)	18 (20)	
≥50%	34 (60)	23 (40)	

*Low-risk patients defined as endometrioid histology and grade 1 or grade 2 disease on preoperative curettage.

**Endometrioid included only.

^#^P-values are calculated by Chi-square test or Fisher exact test.

Low = 1. and 2. tertile

High = 3. tertile

FIGO: International Federation of Gynecology and Obstetrics

### Plasma GDF-15 does not distinguish between hyperplasias and endometrial cancer

**Fig 2:**
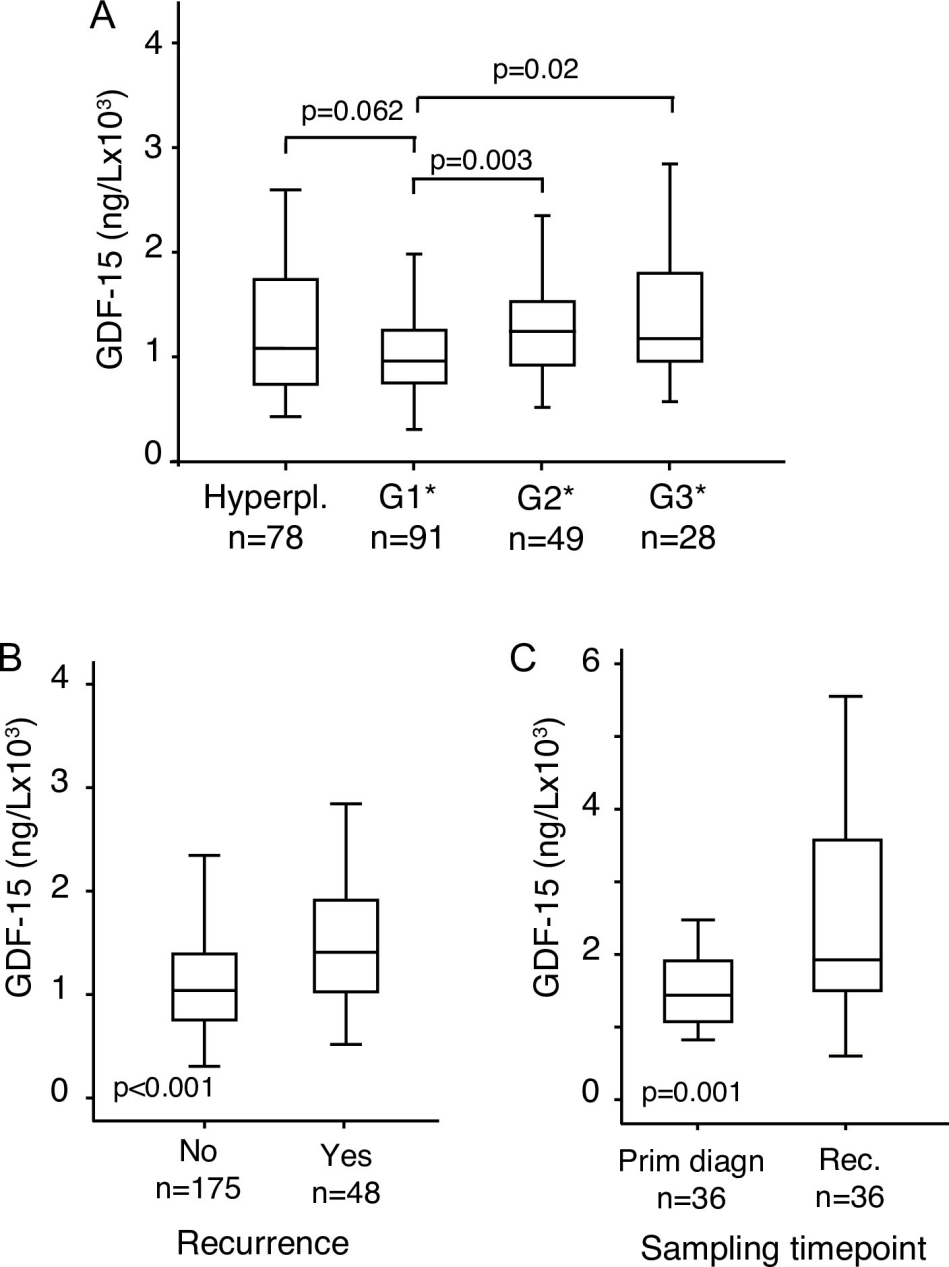
Box plots showing plasma level of GDF-15 in hyperplasias and grade 1–3 (A), in patients who experienced recurrence during their follow-up and in patients who did not (B) and plasma level of GDF-15 in paired samples at time of primary diagnosis and at time of recurrence (C). Number of cases are given. P-values are calculated by the Mann Whitney U test in independent samples and Wilcoxon Signed Rank test in related samples. *Endometrioid included only.

As the plasma level of GDF-15 has been reported to be elevated in cancers compared to healthy controls [20], we investigated if plasma GDF-15 also is a marker for progression from hyperplasia to endometrial cancer. Plasma concentrations of GDF-15 were compared between 78 patients with endometrial hyperplasias and 235 patients with primary endometrial cancer. There was no significant difference between plasma levels of GDF-15 in endometrial hyperplasias (1502 ng/L; 95% CI, 1219–1785 ng/L) and primary tumors (1611 ng/L; 95% CI, 1446–1776 ng/L) (p = 0.807). When performing a more detailed analysis of endometrioid endometrial cancers only, we observed that the increase of GDF-15 occurs between grade 1 and grade 2 (p = 0.003, Fig 2A).

### Elevated plasma level of GDF-15 is associated with recurrent disease

Given the association with aggressive disease, we investigated if GDF-15 could be a marker for recurrence. When analyzing blood samples from endometrial cancer patients at time of primary treatment, the preoperative level of plasma GDF-15 was significantly higher for patients who later experienced recurrence (1780 ng/L; 95% CI; 518–9475 ng/L) than for patients who did not develop recurrent disease (1236 ng/L; 95% CI; 307–7030 ng/L) (p <0.001, Fig 2B). This might indicate a potential for GDF-15 in predicting recurrence. To investigate this further, plasma samples were collected at time of recurrence from 36 patients, and compared to corresponding plasma samples collected at time of primary treatment. For these patients, with available paired samples, plasma levels of GDF-15 at recurrence (2818 ng/L, 95% CI 2088–3548 ng/L) were significantly higher than plasma levels of GDF-15 measured at time of primary diagnosis (1857 ng/L, 95% CI; 1317–2398 ng/L) (p = 0.001, Fig 2C). This may suggest a role for GDF-15 in monitoring recurrence.

### Plasma GDF-15 independently predicts lymph node metastases and recurrence

A prediction model for recurrence was calculated from our merged cohort of 603 patients (described in materials). The cut-off value grouping high and low GDF-15 was 1418 ng/L. Patients with high plasma level of GDF-15 had significantly higher risk of recurrent disease (OR = 3.14; 95% CI 2.10–4.76) in univariate analysis. In the multivariate model, after adjusting for age, postoperative histology and depth of myometrial infiltration, the predictive value remained significant with an adjusted OR of 1.99 (95% CI 1.23–3.22, Table 3). Furthermore, in the merged cohort of 495 patients with lymph node status (also described in materials and cut-off value of 1418 ng/L), high plasma level of GDF-15 was significantly associated with lymph node metastases (OR = 2.64; 95% CI 1.52–4.61) in univariate analysis. In multivariate analysis, adjusting for preoperative histological risk (high; endometrioid grade 3 or non-endometrioid versus low; endometrioid grade 1 or 2) the predictive value of GDF-15 remained significant with an adjusted OR of 2.49 (95% CI 1.42–4.37, Table 4). Age was not significant in in univariate analysis and was therefore not included in the multivariate analysis. Statistical power was too low to perform the analysis in the independent cohort of 235 patients.

**Table 3:** Prediction of recurrence in 603 patients with endometrial cancer, univariate and multivariate logistic regression.

Variable	N	Univariate OR	95% CI	P	Multivariate OR	95% CI	P
**Age**				<0.001			0.043
	603	1.05	1.03–1.07		1.02	1.00–1.05	
**Histology**				<0.001			<0.001
Endometrioid	492						
Non-endometrioid	111	4.50	2.86–7.09		3.72	2.29–6.05	
**Myometrial infiltration**							<0.001
<50%	390			<0.001			
≥50%	213	2.67	1.77–4.04		2.27	1.45–3.55	
**GDF-15**				<0.001			0.005
Low	419						
High tertile	184	3.14	2.07–4.76		1.99	1.23–3.22	

Variables significant in univariate analyses were used in the final multivariate model.

**Table 4:** Prediction of lymph node metastases in 495 patients with endometrial cancer, univariate and multivariate logistic regression.

Variable	N	Univariate OR	95% CI	P	Multivariate OR	95% CI	P
**Curretage histology**				0.014			0.036
Low risk*	373						
High risk**	122	2.06	1.16–3.66		1.87	1.04–3.37	
**GDF-15**				0.001			0.001
Low	346						
High tertile	149	2.64	1.52–4.61		2.49	1.42–4.37	

Variables significant in univariate analyses were used in the final multivariate model.

*Low-risk patients defined as endometrioid histology and grade 1 or grade 2 disease on preoperative curettage.

**High-risk patients defined as endometrioid histology and grade 3 disease or non-endometrioid histology on preoperative curettage.

### High tumor volume detected by MRI is associated with high plasma GDF-15

In order to further validate GDF-15 as a preoperative marker of prognosis, we compared plasma levels of GDF-15 with imaging variables from routine preoperative MRI (n = 141). Patients with high plasma level of GDF-15 had significantly larger tumor volume; with mean tumor size of 17 ml (95% CI: 12–22 ml) in patients with low GDF-15 as opposed to mean tumor size of 27 ml (95% CI: 13–40 ml) in patients with high GDF-15 (p = 0.008). Also, high plasma level of GDF-15 was associated with MRI assessed deep myometrial infiltration (p = 0.05) and cervical stroma invasion (p = 0.03, Table 5).

**Table 5:** Clinical characteristics on preoperative MRI in 141 patients in relation to plasma level of GDF-15.

	Low n (%)	High n (%)	P- value*
**Myometrial infiltration**			0.05
<50%	63 (85)	11 (15)	
≥50%	48 (72)	19 (28)	
**Cervical stroma affection**			0.03
no	90 (84)	17 (16)	
yes	7 (58)	5 (42)	

*P-values are calculated by Chi-Square test.

Low = 1. and 2. tertile

High = 3. tertile

## Discussion

Biomarkers derived from blood samples are easily available and has been less explored compared to tissue biomarkers in endometrial cancer. GDF-15 in plasma has previously been proposed as a biomarker in endometrial cancer [20], and has also been suggested as a serum biomarker in patients with prostate, pancreatic and colon cancers [13, 14, 16]. In addition, GDF-15 has been proposed as a general predictor of cardiovascular disease [23] also in apparently healthy women [24]. In this study, we validate that high level of plasma GDF-15 is associated with clinical characteristics depicting aggressive disease and poor survival in endometrial cancer. In previous studies by Staff et al. elevated plasma level of GDF-15 was associated with aggressive histologic types, lymph node metastases, reduced recurrence-free survival, and death due to endometrial cancer [20]. Our results are in line with these findings and validate GDF-15 as a prognostic marker in endometrial carcinoma.

Plasma biomarkers might be useful for screening if a marker could detect early stages of disease. However, we did not find that plasma levels of GDF-15 distinguish between hyperplasias and grade 1 endometrioid endometrial cancers. Previous reports have identified an increase in plasma GDF-15 from healthy controls to cancer [14, 16, 20]. It is interesting that hyperplasias show similarly high plasma GDF-15 compared to grade 1 endometrioid endometrial cancers. This may indicate that elevation of GDF-15 is an early event and occurs simultaneously with development of hyperplasias. It has previously been reported that serum GDF-15 levels progressively increase from premalignant colonic lesions to cancer initiation with a further increase of plasma levels GDF-15 at time of metastasis [16]. It is known that GDF-15 can induce various pleiotropic effects during cancer progression by negatively or positively modulating cell proliferation, differentiation, apoptosis, invasion, and metastases, dependent of cancer cell types, disease stage, and tumor microenvironment [25]. However, the function of GDF-15 is not yet fully understood. A more thorough investigation of GDF-15 in early stages of disease should include large cohorts of controls and hyperplasias, preferentially also with repeated sampling of individual patients.

For endometrial cancer, there is a need to preoperatively identify patients with aggressive disease to stratify for optimal treatment. Presently, preoperative diagnosis relies on risk classification based on a preoperative biopsy and at some centers, preoperative imaging, preferentially MRI, is included. Addition of an easily obtainable serum biomarker could add relevant information. Importantly, we find that in patients with putative low risk based on preoperative histology (endometrioid grade 1 or 2), high level of GDF-15 predicts poor prognosis and is associated with aggressive features in endometrial cancer. In contrast, low-risk patients with low levels of GDF-15 have a 5-year survival of 97%. This suggests a promising role for GDF-15 in confirming preoperatively that some putative low-risk patients have an excellent prognosis, supporting the clinical value of plasma GDF-15 in endometrial cancer treatment.

Additionally important for treatment of endometrial cancer patients is the ability to identify patients with risk of recurrence. We here find that in preoperative samples, plasma levels of GDF-15 are higher for patients that later experience recurrent disease compared to patients that do not experience recurrence. Further analyses showed the same for paired samples, where level of GDF-15 increased in samples from primary treatment to samples obtained at time of recurrence. When adjusting for age, histology and myometrial infiltration, high plasma level of GDF-15 was an independent marker for predicting recurrence. To the best of our knowledge we demonstrate for the first time that elevated levels of GDF-15 may be helpful in follow-up of patients to detect recurrence. Although the sample size of 36 paired samples is low, the results are promising and indicating a role for GDF-15 in monitoring recurrence. The increase of GDF-15 in aggressive disease and recurrence has been reported in gene- expression signatures from circulating tumor cells [26], further emphasizing our findings. Measuring GDF-15 in plasma from patients with endometrial cancer may be helpful when selecting women who are likely to profit from adjuvant therapy after primary treatment. Monitoring plasma levels of GDF-15 during follow-up can potentially also guide the recommended frequency of follow-up examinations including diagnostic imaging such as MRI or Computer Tomography (CT) after primary treatment.

Interestingly, GDF-15 was superior to histological risk classification in predicting metastatic lymph nodes. GDF-15 could therefore be helpful as an indicator of lymph node metastases and when selecting women for lymphadenectomy. The value of lymph node sampling is controversial and studies are not convincing when evaluating survival benefit and short- and long term complications for the patients who undergo lymphadenectomy [27]. Thus, markers for better prediction of lymph node metastasis may be valuable in the clinic. Novel techniques, such as sentinel lymph node mapping in endometrial carcinoma is increasingly acknowledged [28], however it has limitations among others in relation to obese patients and is still not implemented in most countries, thus a marker in blood samples is still clinically relevant.

Using imaging methods as an adjunct to preoperative serum or tumor biopsy risk stratification may be a useful clinical tool. We demonstrated correlation between plasma GDF-15 and MRI determined tumor size and cervical infiltration. MRI has been reported to outperform that of endocervical curettage for preoperative prediction of cervical stromal invasion [29]. Also, for differentiation of low grade endometrial cancer from endometrial hyperplasia, preoperative MRI and FDG-PET yield promising imaging markers [30]. Imaging markers may thus be better than plasma GDF-15 in detecting early disease since the elevation of GDF-15 seems to occur prior to cancer development. However, the patient population with available imaging data should be larger to validate these findings.

Endometrial cancer is associated with both obesity and high age, factors known to increase the risk of comorbidity such as cardiovascular disease. Cardiovascular disease is not systematically reported in our cohort, and could potentially have biased our results due to its association with high plasma GDF-15 [23, 24]. However, the use of disease-specific survival in our survival analyses and the identified association with both high grade and myometrial infiltration, which is independent of comorbidity and high age, support that GDF-15 specifically detects aggressive endometrial cancer in our cohort. Also, given the association that high plasma level of GDF-15 decreases time to recurrence, further emphasizes that GDF-15 is increased due to the patient’s cancer status as reduced time to recurrence is not likely to be influenced by cardiovascular disease.

Few biomarkers have so far been identified from plasma [2–4] and blood derived markers would be useful in clinical practice as they are less invasive for the patient, are relatively inexpensive and may prove helpful both for preoperative prognostication and in detecting recurrent disease during follow-up. Monitoring plasma levels of GDF-15 during follow-up can potentially guide clinicians when to refer patients to renewed diagnostic imaging by MRI and Computer Tomography (CT) or FDG PET-CT to detect recurrence. Robust plasma markers could thus represent a valuable tool in clinical practice. However, further and larger studies are needed to further evaluate GDF-15 as a plasma marker for recurrence.

We conclude that elevated levels of plasma GDF-15 is associated with an aggressive clinical phenotype and lymph node metastasis in endometrial carcinomas. Elevated GDF-15 is also a marker of recurrent disease at time of recurrence. The clinical value of plasma GDF-15 for prediction of lymph node metastases, prognostication and as a marker of recurrent disease, however, needs to be validated in larger patient cohorts and in clinical trials prior to potential implementation in the clinic.

## References

[1] Amant F, Moerman P, Neven P, Timmerman D, Van Limbergen E, Vergote I. Endometrial cancer. Lancet. 2005;366(9484):491–505. Epub 2005/08/09. 10.1016/S0140-6736(05)67063-8 . 16084259

[2] Tangen IL, Kopperud RK, Visser NC, Staff AC, Tingulstad S, Marcickiewicz J, et al Expression of L1CAM in curettage or high L1CAM level in preoperative blood samples predicts lymph node metastases and poor outcome in endometrial cancer patients. Br J Cancer. 2017;117(6):840–7. Epub 2017/07/29. 10.1038/bjc.2017.235 28751757 PMC5589986

[3] Knific T, Vouk K, Smrkolj S, Prehn C, Adamski J, Rizner TL. Models including plasma levels of sphingomyelins and phosphatidylcholines as diagnostic and prognostic biomarkers of endometrial cancer. J Steroid Biochem Mol Biol. 2018;178:312–21. Epub 2018/01/24. 10.1016/j.jsbmb.2018.01.012 . 29360580

[4] Baydar T, Yuksel O, Sahin TT, Dikmen K, Girgin G, Sipahi H, et al Neopterin as a prognostic biomarker in intensive care unit patients. J Crit Care. 2009;24(3):318–21. Epub 2009/03/31. 10.1016/j.jcrc.2008.06.013 . 19327301

[5] Bootcov MR, Bauskin AR, Valenzuela SM, Moore AG, Bansal M, He XY, et al MIC-1, a novel macrophage inhibitory cytokine, is a divergent member of the TGF-beta superfamily. Proc Natl Acad Sci U S A. 1997;94(21):11514–9. Epub 1997/10/23. 9326641 10.1073/pnas.94.21.11514 PMC23523

[6] Zimmers TA, Jin X, Hsiao EC, McGrath SA, Esquela AF, Koniaris LG. Growth differentiation factor-15/macrophage inhibitory cytokine-1 induction after kidney and lung injury. Shock. 2005;23(6):543–8. Epub 2005/05/18. . 15897808

[7] Bauskin AR, Brown DA, Kuffner T, Johnen H, Luo XW, Hunter M, et al Role of macrophage inhibitory cytokine-1 in tumorigenesis and diagnosis of cancer. Cancer Res. 2006;66(10):4983–6. Epub 2006/05/19. 10.1158/0008-5472.CAN-05-4067 . 16707416

[8] Wollert KC, Kempf T, Peter T, Olofsson S, James S, Johnston N, et al Prognostic value of growth-differentiation factor-15 in patients with non-ST-elevation acute coronary syndrome. Circulation. 2007;115(8):962–71. Epub 2007/02/07. 10.1161/CIRCULATIONAHA.106.650846 . 17283261

[9] Kempf T, Bjorklund E, Olofsson S, Lindahl B, Allhoff T, Peter T, et al Growth-differentiation factor-15 improves risk stratification in ST-segment elevation myocardial infarction. Eur Heart J. 2007;28(23):2858–65. Epub 2007/11/06. 10.1093/eurheartj/ehm465 . 17977844

[10] Lawton LN, Bonaldo MF, Jelenc PC, Qiu L, Baumes SA, Marcelino RA, et al Identification of a novel member of the TGF-beta superfamily highly expressed in human placenta. Gene. 1997;203(1):17–26. Epub 1998/01/13. . 9426002 10.1016/s0378-1119(97)00485-x

[11] Tong S, Marjono B, Brown DA, Mulvey S, Breit SN, Manuelpillai U, et al Serum concentrations of macrophage inhibitory cytokine 1 (MIC 1) as a predictor of miscarriage. Lancet. 2004;363(9403):129–30. Epub 2004/01/17. 10.1016/S0140-6736(03)15265-8 . 14726168

[12] Sugulle M, Dechend R, Herse F, Weedon-Fekjaer MS, Johnsen GM, Brosnihan KB, et al Circulating and placental growth-differentiation factor 15 in preeclampsia and in pregnancy complicated by diabetes mellitus. Hypertension. 2009;54(1):106–12. Epub 2009/05/28. 10.1161/HYPERTENSIONAHA.109.130583 19470878 PMC4167791

[13] Brown DA, Stephan C, Ward RL, Law M, Hunter M, Bauskin AR, et al Measurement of serum levels of macrophage inhibitory cytokine 1 combined with prostate-specific antigen improves prostate cancer diagnosis. Clin Cancer Res. 2006;12(1):89–96. Epub 2006/01/07. 10.1158/1078-0432.CCR-05-1331 . 16397029

[14] Koopmann J, Buckhaults P, Brown DA, Zahurak ML, Sato N, Fukushima N, et al Serum macrophage inhibitory cytokine 1 as a marker of pancreatic and other periampullary cancers. Clin Cancer Res. 2004;10(7):2386–92. Epub 2004/04/10. . 15073115 10.1158/1078-0432.ccr-03-0165

[15] de Wit NJ, Rijntjes J, Diepstra JH, van Kuppevelt TH, Weidle UH, Ruiter DJ, et al Analysis of differential gene expression in human melanocytic tumour lesions by custom made oligonucleotide arrays. Br J Cancer. 2005;92(12):2249–61. Epub 2005/05/19. 10.1038/sj.bjc.6602612 15900300 PMC2361822

[16] Brown DA, Ward RL, Buckhaults P, Liu T, Romans KE, Hawkins NJ, et al MIC-1 serum level and genotype: associations with progress and prognosis of colorectal carcinoma. Clin Cancer Res. 2003;9(7):2642–50. Epub 2003/07/12. . 12855642

[17] Johnen H, Lin S, Kuffner T, Brown DA, Tsai VW, Bauskin AR, et al Tumor-induced anorexia and weight loss are mediated by the TGF-beta superfamily cytokine MIC-1. Nat Med. 2007;13(11):1333–40. Epub 2007/11/06. 10.1038/nm1677 . 17982462

[18] Staff AC, Bock AJ, Becker C, Kempf T, Wollert KC, Davidson B. Growth differentiation factor-15 as a prognostic biomarker in ovarian cancer. Gynecol Oncol. 2010;118(3):237–43. Epub 2010/06/26. 10.1016/j.ygyno.2010.05.032 . 20576287

[19] Trovik J, Salvesen HB, Cuppens T, Amant F, Staff AC. Growth differentiation factor-15 as biomarker in uterine sarcomas. Int J Gynecol Cancer. 2014;24(2):252–9. Epub 2014/01/10. 10.1097/IGC.0000000000000037 . 24401984

[20] Staff AC, Trovik J, Eriksson AG, Wik E, Wollert KC, Kempf T, et al Elevated plasma growth differentiation factor-15 correlates with lymph node metastases and poor survival in endometrial cancer. Clin Cancer Res. 2011;17(14):4825–33. Epub 2011/05/28. 10.1158/1078-0432.CCR-11-0715 . 21616994

[21] Kempf T, Horn-Wichmann R, Brabant G, Peter T, Allhoff T, Klein G, et al Circulating concentrations of growth-differentiation factor 15 in apparently healthy elderly individuals and patients with chronic heart failure as assessed by a new immunoradiometric sandwich assay. Clin Chem. 2007;53(2):284–91. Epub 2006/12/23. 10.1373/clinchem.2006.076828 . 17185363

[22] Haldorsen IS, Gruner R, Husby JA, Magnussen IJ, Werner HM, Salvesen OO, et al Dynamic contrast-enhanced MRI in endometrial carcinoma identifies patients at increased risk of recurrence. Eur Radiol. 2013;23(10):2916–25. Epub 2013/06/05. 10.1007/s00330-013-2901-3 . 23732687

[23] Wiklund FE, Bennet AM, Magnusson PK, Eriksson UK, Lindmark F, Wu L, et al Macrophage inhibitory cytokine-1 (MIC-1/GDF15): a new marker of all-cause mortality. Aging Cell. 2010;9(6):1057–64. Epub 2010/09/22. 10.1111/j.1474-9726.2010.00629.x 20854422 PMC4139960

[24] Brown DA, Breit SN, Buring J, Fairlie WD, Bauskin AR, Liu T, et al Concentration in plasma of macrophage inhibitory cytokine-1 and risk of cardiovascular events in women: a nested case-control study. Lancet. 2002;359(9324):2159–63. Epub 2002/07/02. 10.1016/S0140-6736(02)09093-1 . 12090982

[25] Mimeault M, Batra SK. Divergent molecular mechanisms underlying the pleiotropic functions of macrophage inhibitory cytokine-1 in cancer. J Cell Physiol. 2010;224(3):626–35. Epub 2010/06/26. 10.1002/jcp.22196 20578239 PMC2932466

[26] Alonso-Alconada L, Muinelo-Romay L, Madissoo K, Diaz-Lopez A, Krakstad C, Trovik J, et al Molecular profiling of circulating tumor cells links plasticity to the metastatic process in endometrial cancer. Mol Cancer. 2014;13:223 Epub 2014/09/30. 10.1186/1476-4598-13-223 25261936 PMC4190574

[27] group As, Kitchener H, Swart AM, Qian Q, Amos C, Parmar MK. Efficacy of systematic pelvic lymphadenectomy in endometrial cancer (MRC ASTEC trial): a randomised study. Lancet. 2009;373(9658):125–36. Epub 2008/12/17. 10.1016/S0140-6736(08)61766-3 19070889 PMC2646126

[28] Tanner E, Puechl A, Levinson K, Havrilesky LJ, Sinno A, Secord AA, et al Use of a novel sentinel lymph node mapping algorithm reduces the need for pelvic lymphadenectomy in low-grade endometrial cancer. Gynecol Oncol. 2017;147(3):535–40. Epub 2017/10/24. 10.1016/j.ygyno.2017.10.020 . 29056441

[29] Haldorsen IS, Berg A, Werner HM, Magnussen IJ, Helland H, Salvesen OO, et al Magnetic resonance imaging performs better than endocervical curettage for preoperative prediction of cervical stromal invasion in endometrial carcinomas. Gynecol Oncol. 2012;126(3):413–8. Epub 2012/05/23. 10.1016/j.ygyno.2012.05.009 . 22609110

[30] Berg A, Gulati A, Ytre-Hauge S, Fasmer KE, Mauland KK, Hoivik EA, et al Preoperative imaging markers and PDZ-binding kinase tissue expression predict low-risk disease in endometrial hyperplasias and low grade cancers. Oncotarget. 2017;8(40):68530–41. Epub 2017/10/06. 10.18632/oncotarget.19708 28978135 PMC5620275

